# The neutrophil-to-apolipoprotein A1 ratio is associated with adverse outcomes in patients with acute decompensated heart failure at different glucose metabolic states: a retrospective cohort study

**DOI:** 10.1186/s12944-024-02104-1

**Published:** 2024-04-22

**Authors:** Weimeng Cheng, Tianyue Li, Xiaohan Wang, Tingting Xu, Ying Zhang, Jianzhou Chen, Zhonghai Wei

**Affiliations:** Department of Cardiology, Nanjing Drum Tower Hospital, Affiliated Hospital of Medical School, Nanjing University, Nanjing, 210008 China

**Keywords:** Neutrophil-to-apolipoprotein A1 ratio, Acute decompensated heart failure, Diabetes mellitus, Inflammation, Adverse outcomes

## Abstract

**Background:**

The present study was performed to assess the association between the neutrophil-to-apolipoprotein A1 ratio (NAR) and outcomes in patients with acute decompensated heart failure (ADHF) at different glucose metabolism states.

**Methods:**

We recruited 1233 patients with ADHF who were admitted to Nanjing Drum Tower Hospital, Affiliated Hospital of Medical School, Nanjing University from December 2014 to October 2019. The endpoints were defined as composites of cardiovascular death, nonfatal myocardial infarction, nonfatal ischemic stroke and exacerbation of chronic heart failure. The restricted cubic spline was used to determine the best cutoff of NAR, and patients were divided into low and high NAR groups. Kaplan-Meier plots and multivariable Cox proportional hazard models were used to investigate the association between NAR and the risk of adverse outcomes.

**Results:**

During the five-year follow-up period, the composite outcome occurred in 692 participants (56.1%). After adjusting for potential confounding factors, a higher NAR was associated with a higher incidence of composite outcomes in the total cohort (Model 1: HR = 1.42, 95% CI = 1.22–1.65, *P*<0.001; Model 2: HR = 1.29, 95% CI = 1.10–1.51, *P* = 0.002; Model 3: HR = 1.20, 95% CI = 1.01–1.42, *P* = 0.036). At different glucose metabolic states, a high NAR was associated with a high risk of composite outcomes in patients with diabetes mellitus (DM) (Model 1: HR = 1.54, 95% CI = 1.25–1.90, *P*<0.001; Model 2: HR = 1.40, 95% CI = 1.13–1.74, *P* = 0.002; Model 3: HR = 1.31, 95% CI = 1.04–1.66, *P* = 0.022), and the above association was not found in patients with prediabetes mellitus (Pre-DM) or normal glucose regulation (NGR) (both *P*>0.05).

**Conclusions:**

The NAR has predictive value for adverse outcomes of ADHF with DM, which implies that the NAR could be a potential indicator for the management of ADHF.

## Introduction

It is reported that acute decompensated heart failure (ADHF) is not only the main cause of hospitalization among patients over 65 years old, but also has the highest rate of 30-day readmission among all diseases [1, 2]. In contrast to the substantial improvements in the treatment of chronic HF, little progress has been made in the treatment of ADHF with the 1-year mortality rate reaching 30% [3, 4]. Due to the need for repeated hospitalizations and long-term treatment, ADHF imposes a considerable burden on patients themselves and the health care system, and is likely to become a greater and more serious challenge in the future [5].

Inflammatory reactions are typically involved in various cardiovascular diseases, such as HF, myocardial infarction (MI), atrial fibrillation and atherosclerosis [6–9]. For most patients with HF, systemic congestion is the most prominent feature [10]. Congestion itself leads to the activation of endothelial cells, further aggravating the pro-inflammatory environment [11, 12]. Although neutrophils are vital for host defense, they are also notorious for causing aseptic inflammatory damage [13]. It has been reported that neutrophil counts are independently correlated with HF after MI [14, 15]. Large amounts of neutrophil infiltration were also found in the failed heart [16]. In addition, high-density lipoprotein (HDL) has attracted much attention because of its various cardioprotective functions [17]. As a major protein of HDL, apolipoprotein A1 (ApoA1) can also have anti-inflammatory and antioxidant effects and can reflect the severity of HF [18, 19]. Emerging research suggests that ApoA1 has more predictive value for cardiovascular diseases than HDL and low-density lipoprotein (LDL) levels [20–22].

The neutrophil-to-apolipoprotein A1 ratio (NAR) is a combination of the above two indices. A recent study showed that NAR was directly associated with a higher risk of all-cause death and cardiovascular death in elderly patients with non-valvular atrial fibrillation [23]. NAR has also been reported to be associated with the poor prognosis of cancers [24, 25]. However, studies on this index are quite limited, and the association between NAR and the prognosis of ADHF remains unclear. As a common comorbidity of HF, diabetes mellitus (DM) creates a chronic and low-grade inflammatory environment that exacerbates the deterioration of cardiac functions. Thus, compared with patients with simple HF, patients with HF complicated by DM have a worse prognosis [26, 27]. Interestingly, type 2 diabetes (T2D) is also related to an increase in circulating neutrophils [28]. Another study also demonstrated that after 72 h of intravenous administration of ApoA1 nanoparticles, the neutrophil counts in the circulation of patients with T2D decreased [29]. Therefore, we conducted a retrospective cohort study to evaluate the relationship between NAR and outcomes in patients with ADHF at different glucose metabolic states.

## Patients and methods

### Study population

**Fig. 1:**
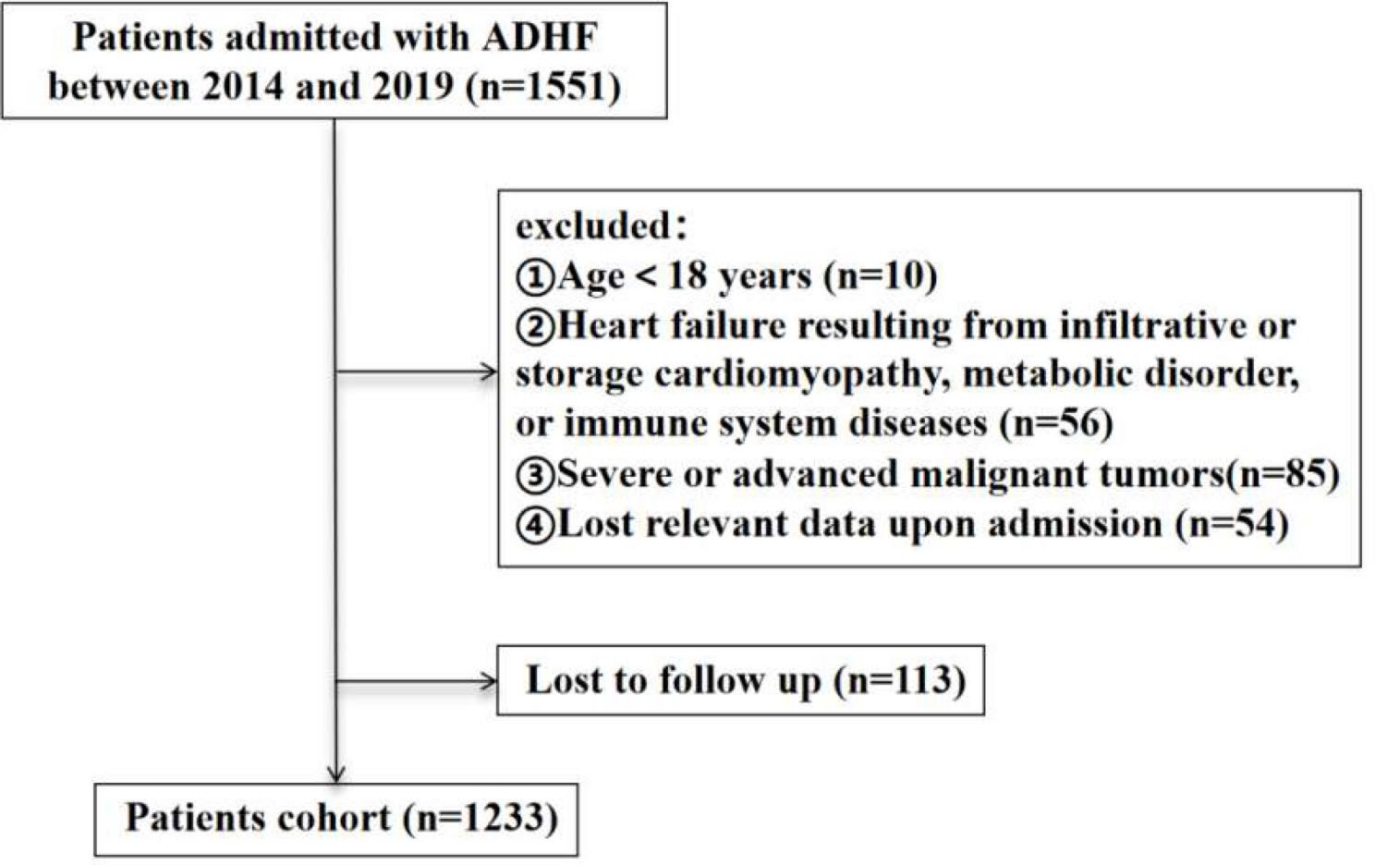
Flow diagram of patient selection

This study included patients with ADHF who were admitted to Nanjing Drum Tower Hospital, Affiliated Hospital of Medical School, Nanjing University from December 2014 to October 2019. It was a single-center retrospective analysis. ADHF was defined according to the criteria established in the 2021 European Society of Cardiology (ESC) diagnostic and treatment guidelines for acute and chronic HF [30]. Among 1551 patients, 318 were excluded due to the following criteria: (1) being under 18 years old; (2) having HF resulting from infiltrative or storage cardiomyopathy, metabolic disorders, or immune system diseases; (3) having severe or advanced malignant tumors; (4) being lost relevant data upon admission; and (5) being lost to follow-up. Ultimately, 1233 patients were enrolled in this study (Fig. 1).

### Data collection

The trained physicians collected the complete patient data through the electronic medical records system. The data included demographic information, clinical history, medication use, echocardiogram results and blood sample test results. The demographic information collected in this study included sex, age, body mass index (BMI), as well as smoking and drinking status. The medical history data included conditions such as glucose metabolic states, hypertension, hyperuricemia, infection, percutaneous coronary intervention (PCI), prior MI, prior stroke, valvular heart disease, atrial fibrillation and anemia. The medications used included calcium channel blockers (CCBs), angiotensin converting enzyme inhibitors (ACEIs)/angiotensin receptor blockers (ARBs), beta blockers, diuretics, digitalis, antiplatelets, anticoagulants, and statins. Echocardiography was performed using a PHILIPS IE 33 real-time three-dimensional color Doppler ultrasonic diagnostic instrument. The echocardiographic results included the left ventricular ejection fraction (LVEF), left ventricular systolic diameter (LVDs), left ventricular diastolic diameter (LVDd) and pulmonary arterial pressure (PAP). The blood sample data comes from fasting venous blood collected within 24 h of hospitalization. The blood was into plastic tubes with or without EDTA. Neutrophil counts, white blood cell counts (WBC), red blood cell counts (RBC), platelet counts (PLT) and hemoglobin (HB) were measured using a Sysmex automated hematology analyzer XE-2100 (Sysmex, Kobe, Japan). ApoA1, apolipoprotein B (ApoB), fasting plasma glucose (FPG), aspartate transaminase (ALT), alanine aminotransferase (AST), total cholesterol, triglyceride, low-density lipoprotein cholesterol (LDL-C), high-density lipoprotein cholesterol (HDL-C), albumin (ALB), C-reactive protein (CRP), uric acid (UA), estimated glomerular filtration rate (eGFR), serum creatinine (Scr), serum potassium and serum sodium were assayed using a HITACHI automatic biochemical analyzer 7600–020 (HITACHI, Tokyo, Japan). The glycated hemoglobin (HbA1c) level was determined using a Tosoh Automated Glycohemoglobin Analyzer (HLC-723G8, Tokyo, Japan). We measured plasma B-type natriuretic peptide (BNP) concentrations using an ELISA kit (Raybiotech, Norcross, GA, USA).

### Definitions

Glucose metabolic states can be categorized into three types: normal glucose regulation (NGR), prediabetes mellitus (Pre-DM), and DM. According to the guideline recommendations of the American Diabetes Association, DM was defined as FPG ≥ 7.0 mmol/L or HbA1c ≥ 6.5%, or currently taking hypoglycemic medications. Pre-DM was defined as 5.6 mmol/L ≤ FPG<7.0 mmol/L, or 5.7%≤HbA1c<6.5%. NGR referred to patients without Pre-DM or DM [31]. NAR was defined as the ratio of the neutrophil count to the ApoA1 concentration (×10^9^/mmoL).

### Endpoints and follow-up

The endpoints were a composite of cardiovascular death, nonfatal myocardial infarction, nonfatal ischemic stroke and exacerbation of chronic heart failure. Cardiovascular death included deaths caused by heart diseases such as myocardial infarction, arrhythmia or HF. Patients were followed up by experienced doctors via telephone and/or outpatient services once every six months.

### Statistical analysis

The restricted cubic spline was used to determine the optimal cutoff value for NAR. Patients were divided into two groups based on the optimal cutoff value: the low NAR group (NAR-L) and the high NAR group (NAR-H). Continuous variables were presented as medians with interquartile ranges (IQRs), and categorical variables were presented as frequencies with percentages. Continuous and categorical variables were compared between groups using the Mann‒Whitney U test, chi-square test or Fisher’s exact test, as appropriate. Kaplan-Meier survival curve was used for survival analysis, and the Log-rank test was used to compare the differences between groups. We used multivariable Cox proportional hazards models to assess the relationships between NAR and the risk of adverse outcomes. Schoenfeld residuals were used to validate the hypothesis of proportional hazards prior to these analyses. We checked for collinearity among independent variables by assessing the variance inflation factor, which was less than 5, indicating the absence of collinearity. We have established three models to adjust for the influence of confounding factors: Model 1, adjusted for sex and age; Model 2, adjusted for Model 1 plus infection, prior MI, hypertension, hyperuricemia, ACEI/ARB, diuretics and statins; and Model 3, adjusted for Model 2 plus LVEF, BNP, HDL-C, CRP, ALB, HB, UA, Scr and serum sodium.

In addition, the prognostic value of the NAR in patients with ADHF at different glucose metabolic states was evaluated using the models described above. The results were reported in terms of hazard ratios (HRs) and 95% confidence intervals (CIs). The NAR-L was used as a reference in the above models. The correlation between NAR and several biomarkers was analyzed by the Spearman correlation coefficients and visualized. Patients with DM were analyzed by subgroup, which included age (>70 years versus ≤ 70 years), sex (male versus female), smoking status (no versus yes), BMI (>24 kg/m2 versus ≤ 24 kg/m2), LVEF (>40% versus ≤ 40%), eGFR (>60 ml/min versus ≤ 60 ml/min), history of hypertension (no versus yes), history of prior stroke (no versus yes), and history of prior MI (no versus yes), and the results are shown as forest plots. We used likelihood ratio tests to test interactions between the subgroups. All the data were analyzed by R 4.3.0 and SPSS 27.0.1, and *P*<0.05 was considered to indicate statistical significance.

## Results

### Baseline characteristics of the study cohort

**Fig. 2:**
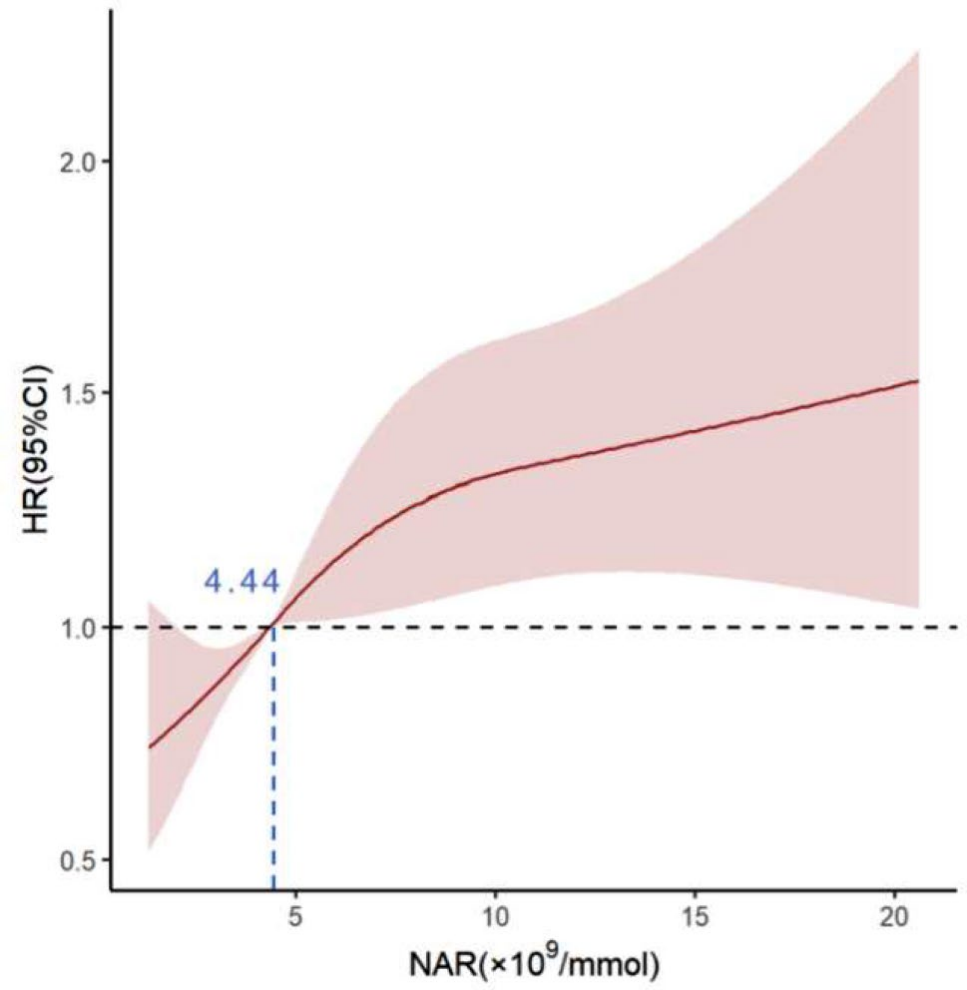
HRs for composite outcomes based on restricted cubic splines for NAR. Red lines represent for hazard ratios, and red areas represent for 95% CIs

The restricted cubic spline analysis showed that the risk of composite endpoints increased linearly as the NAR increased (*P*>0.05 for Non-linear test). The optimal cutoff value of the NAR was determined to be 4.44 (Fig. 2). Patients were divided into two groups according to the cutoff value [NAR-L (NAR<4.44) and NAR-H (NAR ≥ 4.44)]. The baseline data for both groups is presented in Table 1. In the high NAR group, neutrophil counts, HbA1c, FPG, BNP, WBC, PLT, ALT, CRP, UA, Scr, LVDd, LVDs, the proportion of males, infection, prior MI, PCI, hypertension, hyperuricemia, diuretics and statin use were significantly higher. In addition, the levels of ApoA1, TC, LDL-C, HDL-C, ALB, eGFR, LVEF, serum sodium concentration, and the percentage of patients receiving ACEIs/ARBs were significantly lower. It is worth noting that the proportion of patients with DM in the high NAR group was significantly higher than that in the low NAR group, while the proportion of individuals with NGR was significantly lower than that in the low NAR group (all *P*<0.05).

**Table 1:** Clinical characteristics of patients according to the level of NAR

	Total(*n* = 1233)	NAR-L(NAR<4.44) (*n* = 633)	NRA-H(NAR ≥ 4.44) (*n* = 600)	P value
Female, n (%)	540 (43.8)	311 (49.1)	229 (38.2)	< 0.001
Age (years)	73.00 [64.00, 80.00]	74.00 [65.00, 80.00]	73.00 [62.00, 81.00]	0.145
BMI (kg/m2)	23.74 [21.88, 25.56]	23.71 [21.88, 25.39]	23.79 [21.88, 25.88]	0.35
Smoking, n (%)	270 (21.9)	130 (20.5)	140 (23.3)	0.264
Drinking, n (%)	139 (11.3)	73 (11.5)	66 (11.0)	0.837
**Glucose metabolism status, n (%)**				
DM	610 (49.5)	288 (45.5)	322 (53.7)	0.005
Pre-DM	445 (36.1)	238 (37.6)	207 (34.5)	0.283
NGR	178 (14.4)	107 (16.9)	71 (11.8)	0.014
**Medical history, n (%)**				
Hypertension	816 (66.2)	397 (62.7)	419 (69.8)	0.01
Hyperuricemia	535 (43.4)	237 (37.4)	298 (49.7)	< 0.001
Infection	280 (22.7)	90 (14.2)	190 (31.7)	< 0.001
PCI	259 (21.0)	109 (17.2)	150 (25.0)	0.001
Prior MI	171 (13.9)	67 (10.6)	104 (17.3)	0.001
Prior stroke	280 (22.7)	132 (20.9)	148 (24.7)	0.126
Valvular heart disease	197 (16.0)	98 (15.5)	99 (16.5)	0.682
Atrial fibrillation	608 (49.3)	307 (48.5)	301 (50.2)	0.597
Anemia	281 (22.8)	149 (23.5)	132 (22.0)	0.565
**Medications at admission, n (%)**				
CCBs	258 (20.9)	135 (21.3)	123 (20.5)	0.774
ACEIs/ARBs	510 (41.4)	289 (45.7)	221 (36.8)	0.002
Beta blockers	818 (66.3)	406 (64.1)	412 (68.7)	0.105
Diuretics	946 (76.7)	463 (73.1)	483 (80.5)	0.003
Digitalis	237 (19.2)	113 (17.9)	124 (20.7)	0.237
Antiplatelets	594 (48.2)	290 (45.8)	304 (50.7)	0.099
Anticoagulants	346 (28.1)	173 (27.3)	173 (28.8)	0.6
Statins	670 (54.3)	324 (51.2)	346 (57.7)	0.026
LVEF (%)	45.00 [35.00, 54.00]	46.00 [37.00, 54.25]	43.00 [33.00, 52.00]	< 0.001
LVDs (cm)	4.34 [3.70, 5.15]	4.26 [3.67, 4.98]	4.41 [3.73, 5.35]	0.005
LVDd (cm)	5.70 [5.16, 6.40]	5.70 [5.15, 6.27]	5.72 [5.20, 6.50]	0.047
PAP (mmHg)	42.00 [35.00, 50.00]	40.00 [35.00, 50.00]	42.00 [35.00, 50.00]	0.272
NAR (×10^9^/mmol)	4.37 [3.16, 6.07]	3.20 [2.50, 3.86]	6.16 [5.16, 8.30]	< 0.001
Neutrophil counts (×10^9^/L)	4.01 [3.10, 5.23]	3.19 [2.56, 3.80]	5.14 [4.31, 6.59]	< 0.001
ApoA1 (mmol/L)	0.92 [0.77, 1.09]	1.03 [0.89, 1.17]	0.80 [0.69, 0.94]	< 0.001
ApoB (mmol/L)	0.72 [0.56, 0.89]	0.71 [0.55, 0.88]	0.72 [0.58, 0.90]	0.441
HbA1c (%)	6.18 [5.80, 6.80]	6.10 [5.79, 6.68]	6.22 [5.80, 7.00]	0.001
FPG (mmol/L)	5.12 [4.56, 6.18]	5.01 [4.52, 5.77]	5.29 [4.63, 6.69]	< 0.001
BNP (ng/L)	538.00 [235.00, 934.00]	442.00 [201.00, 777.30]	644.00 [318.25, 1242.50]	< 0.001
WBC (×10^9^/L)	6.20 [5.00, 7.70]	5.20 [4.40, 6.10]	7.60 [6.30, 8.90]	< 0.001
RBC (×10^12^/L)	4.25 [3.76, 4.64]	4.24 [3.76, 4.57]	4.25 [3.76, 4.75]	0.065
PLT (×10^9^/L)	167.00 [132.00, 207.00]	157.00 [123.00, 193.00]	179.50 [140.75, 223.00]	< 0.001
HB (g/L)	128.00 [112.00, 141.00]	128.00 [113.00, 140.00]	128.50 [111.00, 142.00]	0.418
ALT (U/L)	19.40 [13.60, 31.00]	18.90 [13.70, 27.80]	20.80 [13.57, 34.40]	0.012
AST (U/L)	23.30 [18.10, 31.70]	23.30 [18.60, 30.40]	23.25 [17.40, 34.00]	0.771
Total cholesterol (mmol/L)	3.63 [3.01, 4.42]	3.81 [3.20, 4.60]	3.44 [2.90, 4.19]	< 0.001
Triglyceride (mmol/L)	1.04 [0.76, 1.44]	1.03 [0.73, 1.46]	1.04 [0.79, 1.43]	0.202
LDL-C (mmol/L)	1.94 [1.48, 2.52]	2.00 [1.51, 2.59]	1.89 [1.44, 2.44]	0.028
HDL-C (mmol/L)	0.96 [0.77, 1.20]	1.10 [0.89, 1.33]	0.85 [0.70, 1.01]	< 0.001
ALB (g/L)	38.20 [35.60, 40.80]	38.80 [36.50, 41.40]	37.20 [34.68, 39.82]	< 0.001
CRP (mg/L)	4.20 [2.40, 9.70]	3.50 [2.10, 5.50]	6.10 [2.90, 20.97]	< 0.001
UA (µmol/L)	428.00 [337.00, 547.00]	406.00 [325.00, 506.00]	454.00 [357.00, 584.00]	< 0.001
Scr (µmol/L)	84.00 [67.00, 110.00]	78.00 [63.00, 99.00]	93.50 [73.00, 121.25]	< 0.001
eGFR (ml/min)	69.00 [47.60, 87.00]	74.00 [54.00, 89.30]	62.00 [42.75, 81.00]	< 0.001
Serum potassium (mmol/L)	3.95 [3.68, 4.25]	3.96 [3.69, 4.20]	3.94 [3.66, 4.30]	0.701
Serum sodium (mmol/L)	141.00 [138.00, 142.90]	141.30 [138.90, 143.20]	140.45 [137.20, 142.60]	< 0.001

### Associations between NAR and endpoints in the total cohort

**Fig. 3:**
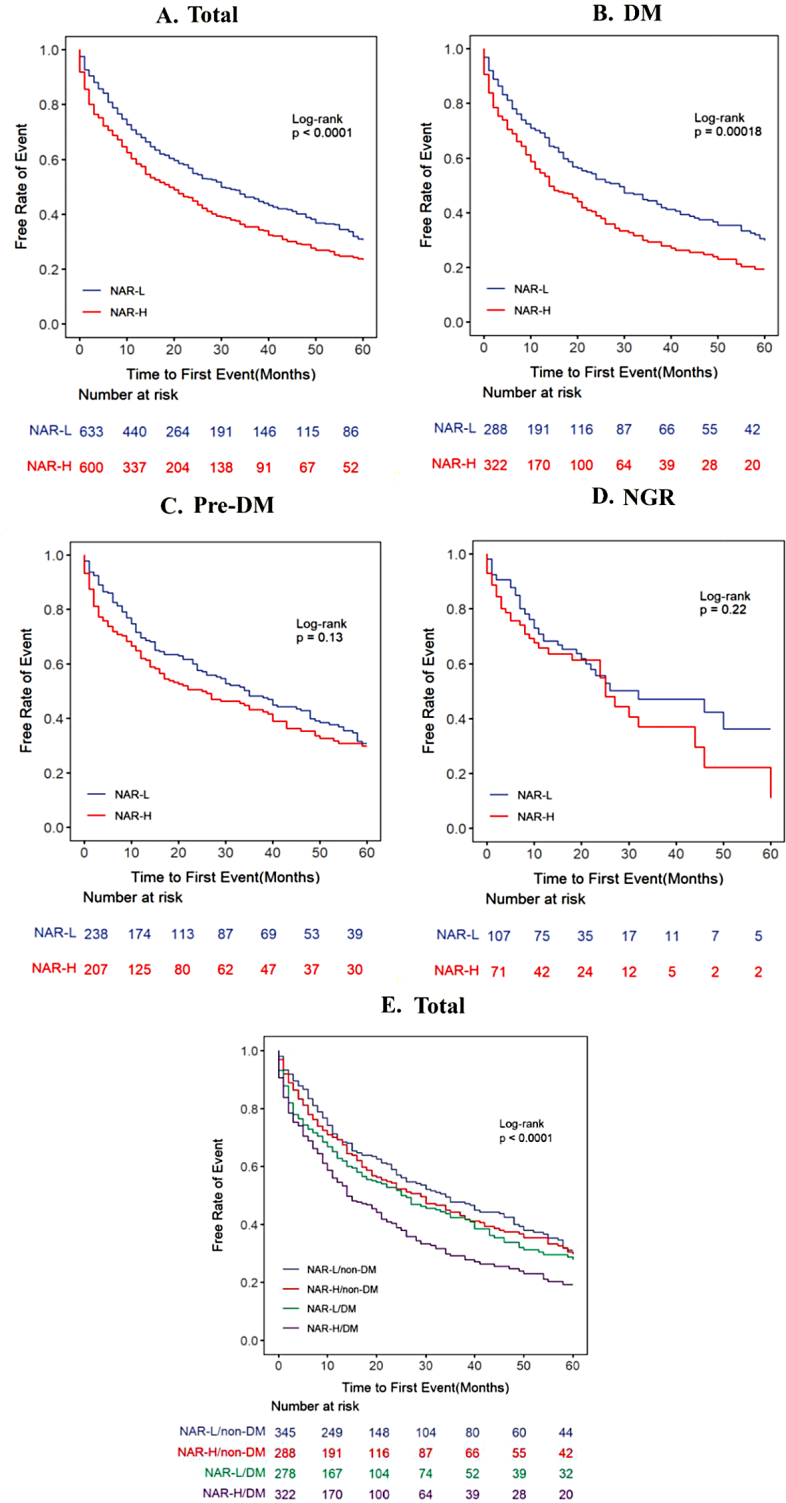
Kaplan-Meier curves for composite outcomes according to the NAR level in total patients (**A**), patients with DM (**B**), patients with Pre-DM (**C**) and patients with NGR (**D**). Kaplan-Meier curves for composite outcomes according to status of both NAR levels and glucose metabolic states **(E**)

During a follow-up period of up to five years, 692 patients (56.1%) developed composite outcomes. The Kaplan-Meier curves (Fig. 3A) revealed that NAR was significantly associated with the incidence of composite outcomes in the total cohort (Log-Rank test *P*<0.0001). The results of the three multivariable Cox proportional hazard models used to test the association between NAR groups and composite outcomes showed in Table 2. Consequently, a higher NAR was associated with a higher incidence of composite outcomes in all patients (Model 1: HR = 1.42, 95% CI = 1.22–1.65, *P*<0.001; Model 2: HR = 1.29, 95% CI = 1.10–1.51, *P* = 0.002; Model 3: HR = 1.20, 95% CI = 1.01–1.42, *P* = 0.036).

### Associations between NAR and endpoints in patients at different glucose metabolic states

At different glucose metabolic states, Kaplan-Meier curves (Fig. 3B–D) indicated that the incidence of the composite endpoints was significantly different between NAR groups in patients with DM (Log-Rank test *P* = 0.00018), whereas no significant distinction was observed in Pre-DM and NGR subgroups (all Log-Rank test *P*>0.05). Meanwhile, Table 2 showed that a high NAR group was associated with a high incidence of composite outcomes in patients with DM, consistently observed across three multivariable Cox models (Model 1: HR = 1.54, 95% CI = 1.25–1.90, *P*<0.001; Model 2: HR = 1.40, 95% CI = 1.13–1.74, *P*=0.002; Model 3: HR = 1.31, 95% CI = 1.04–1.66, *P* = 0.022). In contrast, there were no significant differences between NAR groups in Pre-DM or NGR patients (all *P*>0.05). Compared with other groups, the NAR-H/DM group had the highest risk of developing composite outcomes as illustrated in Fig. 3E (overall Log-Rank test *P*<0.0001; NAR-H/DM and NAR-H/no-DM Log-Rank test *P* = 0.016). Statistical analysis of the Schoenfeld residuals indicated that the proportional hazard assumption was not violated.

**Table 2:** Cox proportional hazard model of NAR and different glucose metabolic states

		Model 1			Model 2		Model 3	
Glucose regulation status	NAR	HR (95% CI)	P value	HR (95% CI)		P value	HR (95% CI)	P value
Total	low	Ref.		Ref.			Ref.	
	high	1.42(1.22–1.65)	<0.001	1.29(1.10–1.51)		0.002	1.20(1.01–1.42)	0.036
DM	low	Ref.		Ref.			Ref.	
	high	1.54(1.25–1.90)	<0.001	1.40(1.13–1.74)		0.002	1.31(1.04–1.66)	0.022
Pre-DM	low	Ref.		Ref.			Ref.	
	high	1.24(0.96–1.59)	0.097	1.06(0.81–1.38)		0.692	1.04(0.78–1.40)	0.78
NGR	low	Ref.		Ref.			Ref.	
	high	1.39(0.88–2.17)	0.154	1.42(0.89–2.27)		0.141	1.09(0.60–1.95)	0.785

*Note* Model 1: adjusted for sex, age; Model 2: adjusted for model 1 plus infection, prior MI, hypertension, hyperuricemia, ACEIs/ARBs, diuretics and statins; Model 3: adjusted for model 2 plus LVEF, BNP, HDL-C, CRP, ALB, HB, UA, Scr and serum sodium

### Correlations between NAR and other biomarkers

**Fig. 4:**
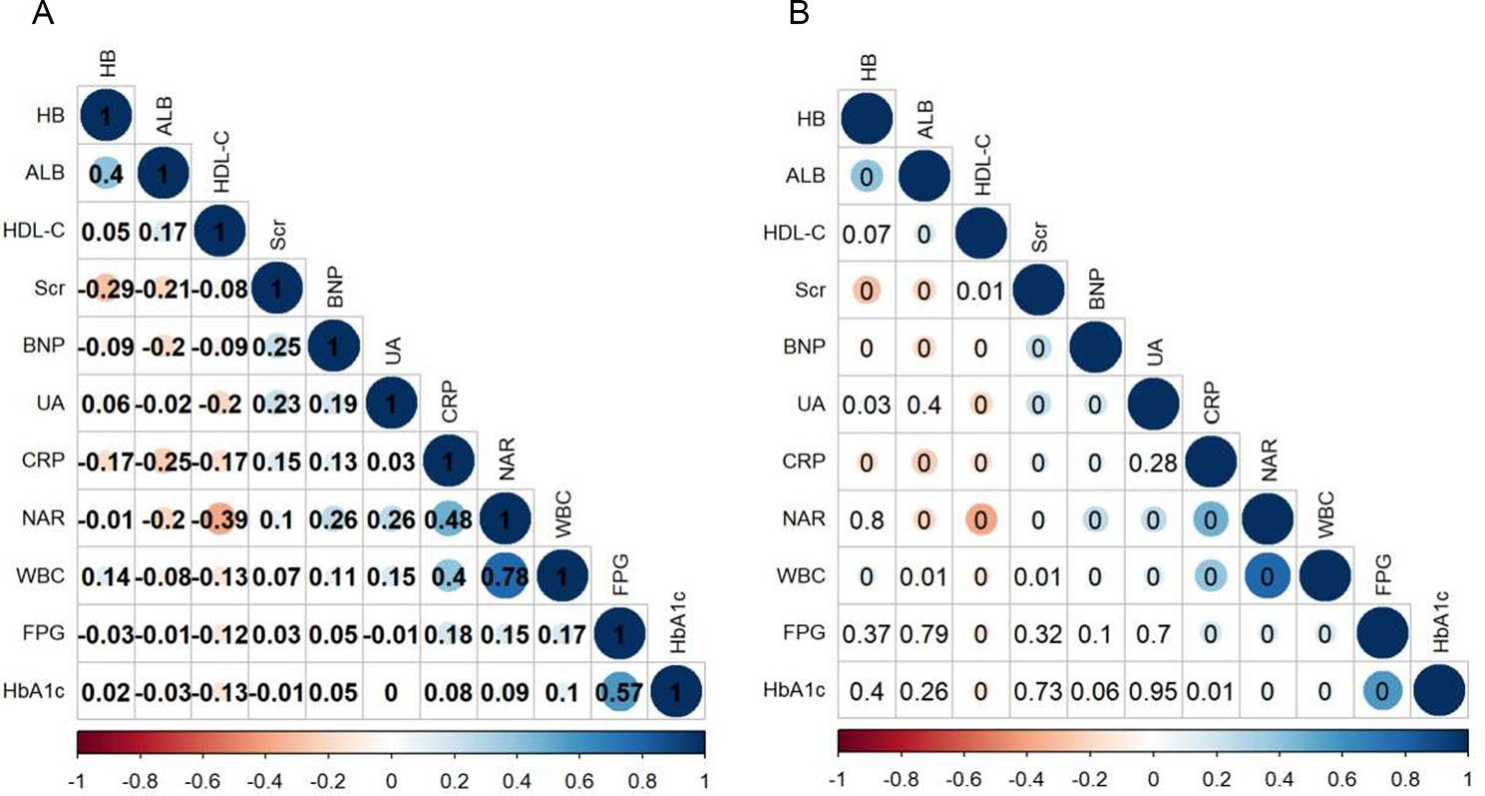
The correlation coefficients **(A)** and P values **(B)** between NAR and other biomarkers

The results of the Spearman correlation coefficients were presented in Fig. 4. Figure 4A displays the correlation coefficients between NAR and several biomarkers, while Fig. 4B presents the P values (0 represents<0.001). A positive correlation was observed between NAR and HbA1c, FPG, CPR, WBC, BNP, UA and Scr, while a negative correlation was observed with HDL-C and ALB (all *P*<0.05).

### Subgroup analysis of patients with DM

**Fig. 5:**
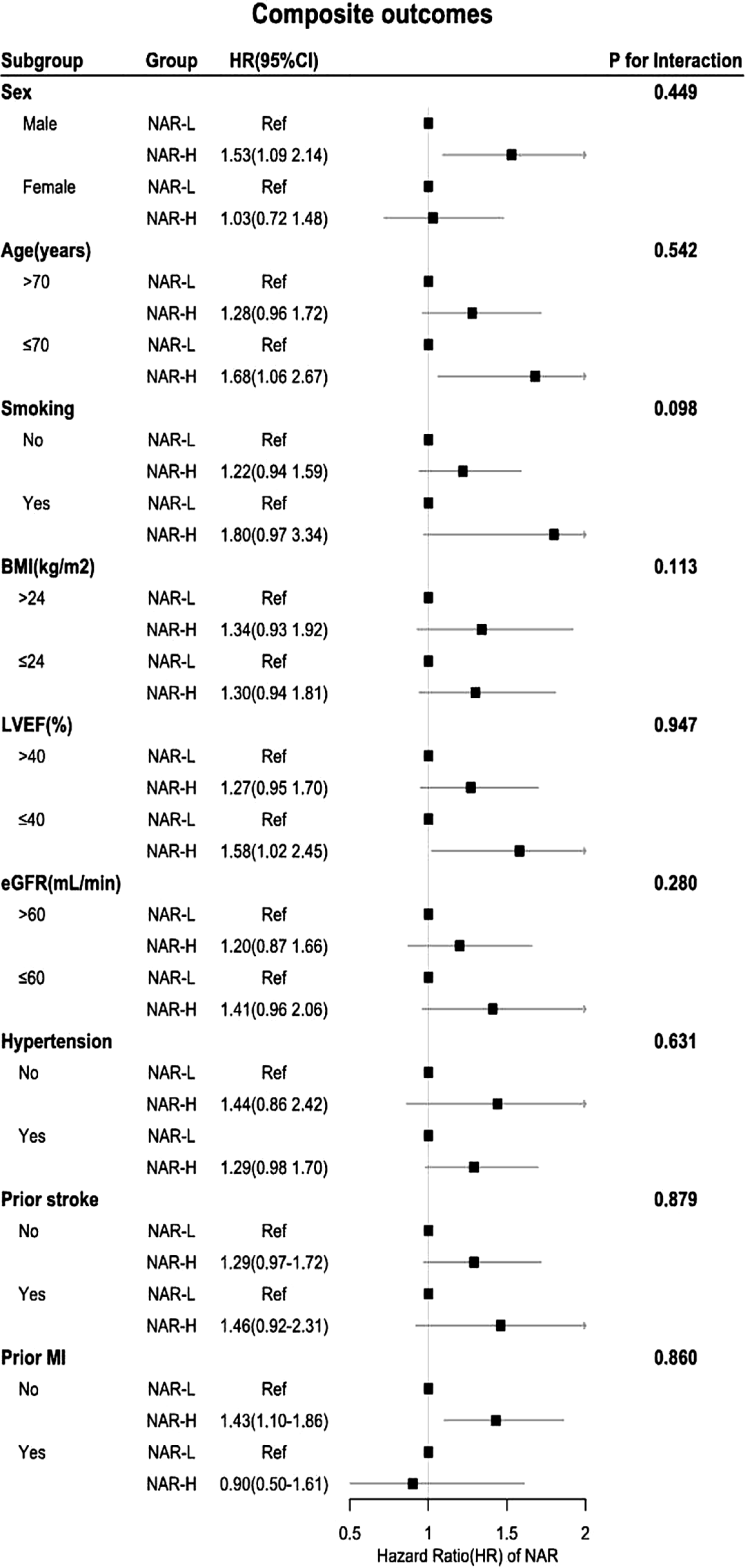
The forest plot of composite outcomes according to subgroups of patients with DM. The adjusted models included sex, age, infection, prior MI, hypertension, hyperuricemia, ACEIs/ARBs, diuretics, statins, LVEF, BNP, HDL-C, CRP, ALB, HB, UA, Scr and serum sodium

In patients with DM, the impact of different NAR levels on the risk of composite outcomes remained consistent across the predefined subgroups (Fig. 5). Furthermore, there were no interactions between NAR and any of the pre-specified variables (all P values for interaction>0.05).

## Discussion

This was the first study to explore the relationship between NAR, a simple and workable indicator, and the risk of adverse outcomes in patients with ADHF. In this retrospective study, NAR was positively correlated with glucose metabolism-related indices such as HbA1c and FPG. Furthermore, a high NAR was significantly associated with an increased risk of adverse outcomes (cardiovascular death, nonfatal myocardial infarction, nonfatal ischemic stroke and exacerbation of chronic heart failure) in patients with ADHF complicated by DM. Nonetheless, we did not find the above association in patients with Pre-DM or NGR.

HF is characterized by low-grade chronic inflammation, regardless of the ejection fraction [32]. As a marker of inflammation, neutrophils are directly related to the severity of HF and plasma CRP levels [33]. In contrast to other cardiovascular diseases, such as nonfatal myocardial infarction and coronary death, neutrophil counts are more strongly associated with the onset of HF [34]. Previous studies have shown that low ApoA1 levels are strongly associated with poor outcomes in patients with left ventricular dysfunction and non-ischemic HF [19, 35]. Furthermore, as one of the serious complications of HF, DM has the ability to induce a systemic inflammatory state. Previous studies have shown that the increase in inflammatory biomarkers observed in HF is mediated not only by HF itself, but also by DM [36, 37]. Patients with T2D had significantly higher levels of peripheral blood leukocytes and neutrophils than those without the disease [38]. Woo et al. demonstrated that the increase in neutrophil counts throughout the body was associated with the presence and severity of DM and diabetic retinopathy [39]. Additionally, a significant difference in plasma lipid composition between diabetic and nondiabetic subjects is the low levels of HDL [40]. Wu et al. pointed out that the low level of ApoA1 was significantly and independently correlated with the development of new T2D in healthy individuals within 4 years [41]. A clinical transformation experiment demonstrated that intravenous injection of ApoA1 nanoparticles in patients with T2D could directly reduce the levels of circulating neutrophils [29]. Previous research has suggested that ApoA1 may have a stronger inhibitory effect on the activation of neutrophils than HDL [42]. Above all, these findings highlight the potential of neutrophils and ApoA1 as effective targets for the treatment of HF and DM. However, it is unclear whether the delivery of ApoA1 in the therapeutic setting of HF is sufficient to modulate the cardiovascular inflammatory response for beneficial results, and more studies are needed to further elaborate on this.

NAR is a metric that combines the above two useful biomarkers of HF. It has been reported that high NAR levels are associated with a high risk of death in elderly patients with non-valvular atrial fibrillation [23]. Meanwhile, NAR could be used to predict overall survival in patients with nasopharyngeal carcinoma and hepatocellular carcinoma [24, 25]. Nonetheless, the association between NAR and outcomes of ADHF has not yet been identified. In the present study, we confirmed the predictive value of NAR for adverse outcomes in patients with ADHF and DM. The potential mechanism underlying the relationship between NAR and ADHF is largely attributed to the biological functions of neutrophils and ApoA1. Activated neutrophils trap and kill bacteria by releasing neutrophil extracellular traps (NETs). NETs are released into the extracellular space, and have high proinflammatory, cytotoxic and thrombogenic effects [43]. An increase in the expression of various molecular signals in HF often leads to an increase in NETs formation (termed NETosis) [13]. Cardiac pressure overload induces NETosis, which leads to significant platelet recruitment and consequently to HF [44]. Hyperglycemia increases the production of reactive oxygen species (ROS) by neutrophils [45]. Excessive ROS induce NETosis in neutrophils, increasing the gene expression and secretion of S100A8/S100A9. This further amplifies myocardial inflammation, promotes myocardial remodeling and heart failure, and ultimately leads to a vicious cycle [45–48].

The anti-inflammatory effects of HDL and ApoA1 are potentially important for preventing cardiovascular events [49]. ApoA1 also has remarkable anti-diabetic properties [50]. Numerous studies have demonstrated that ApoA1 can improve blood sugar control by enhancing insulin sensitivity, and can also increase or inhibit the expression of transcription factors that are essential for β-cell identity and survival to enhance their functions [51–53]. In addition, ApoA1 can inhibit the function of activated neutrophils, which can prevent further damage to organs and tissues [42, 54]. However, the anti-inflammatory properties of HDL and ApoA1 are lost in patients with T2D, which is attributed to the non-enzymatic glycosylation of ApoA1 by reactive α-oxoaldehydes [55, 56]. Notably, in the same hyperglycemic environment, unlike in patients with T2D, the HDL levels are not low in patients with type 1 diabetes (T1D), and the number of circulating neutrophils is slightly reduced [40, 57]. Recently, Huang et al. showed that neutrophil counts decreased in patients with T1D, but the opposite was true in patients with T2D [58]. There are significant differences in neutrophil counts and blood lipid compositions between T1D and T2D, but the relevant mechanisms remain unclear.

In the future, high NAR levels could be used to identify patients at the high risk of adverse outcomes in patients with ADHF complicated by DM. This study has several limitations. First, due to the limitations of the retrospective design, we were unable to dynamically monitor the NAR of patients during follow-up. Second, this was a single-center study with a limited sample size, and data bias cannot be completely avoided, despite adjusting for many confounding factors. The study focused solely on patients with ADHF complicated by T2D. However, the relationship between NAR and T1D still needs to be explored. Moreover, our findings need to be validated in a prospective cohort study.

## Conclusion

This study demonstrated that NAR was independently associated with a poor prognosis in patients with ADHF complicated with DM, but this relationship was not observed in patients with Pre-DM and NGR.

## Data Availability

No datasets were generated or analysed during the current study.

## Abbreviations

ACEIs: angiotensin converting enzyme inhibitors
ARBs: angiotensin receptor blockers
ADHF: acute decompensated heart failure
ALB: albumin
ALT: aspartate transaminase
ApoA1: apolipoprotein A1
ApoB: apolipoprotein B
AST: alanine aminotransferase
BMI: body mass index
BNP: B-type natriuretic peptide
CCBs: calcium channel blockers
CI: confidence interval
CRP: C-creative protein
DM: diabetes mellitus
eGFR: estimated glomerular filtration rate
ESC: European Society of Cardiology
FPG: fasting plasma glucose
HB: hemoglobin
HbA1c: glycosylated hemoglobin
HDL: high-density lipoprotein
HDL-C: high density lipoprotein-cholesterol
HF: heart failure
HR: hazard ratio
IORs: interquartile ranges
LDL: low-density lipoprotein
LDL-C: low density lipoprotein-cholesterol
LVDd: left ventricular diastolic diameter
LVDs: left ventricular systolic diameter
LVEF: left ventricular ejection fraction
MI: myocardial infarction
NAR: Neutrophil-to-apolipoprotein A1 ratio
NETs: neutrophil extracellular traps
NETosis: NETs formation
NGR: normal glucose regulation
PAP: pulmonary arterial pressure
PCI: percutaneous coronary intervention
PLT: platelet counts
Pre-DM: prediabetes mellitus
RBC: red blood cell counts
ROS: reactive oxygen species
Scr: serum creatinine
T1D: type 1 diabetes
T2D: type 2 diabetes
UA: uric acid
WBC: white blood cell counts
